# Towards a New Dawn for Neuro-Oncology: Nanomedicine at the Service of Drug Delivery for Primary and Secondary Brain Tumours

**DOI:** 10.3390/brainsci15020136

**Published:** 2025-01-30

**Authors:** Smita Khilar, Antonina Dembinska-Kenner, Helen Hall, Nikolaos Syrmos, Gianfranco K. I. Ligarotti, Puneet Plaha, Vasileios Apostolopoulos, Salvatore Chibbaro, Giuseppe Maria Vincenzo Barbagallo, Mario Ganau

**Affiliations:** 1 Department of Neurosurgery, Oxford University Hospitals NHS Foundation Trust, Oxford OX3 0AG, UK; smita.khilar@royalberkshire.nhs.uk (S.K.); helen.hall@balliol.ox.ac.uk (H.H.);; 2 School of Medicine, Aristotle University of Thessaloniki, 54124 Thessaloniki, Greece; 3 Aerospace Medical Institute of Milan “A. Mosso”, Italian Air Force, 20138 Milan, Italy; 4 Neurosurgery Unit, Department of Medical and Surgical Sciences and Neurosciences, Siena University, 53100 Siena, Italy; 5 Department of Neurological Surgery, Policlinico “G. Rodolico-S. Marco” University Hospital, 95121 Catania, Italy; 6 Nuffield Department of Clinical Neurosciences, University of Oxford, Oxford OX3 9DU, UK

**Keywords:** brain tumours, gliomas, glioblastoma, meningioma, brain metastases, malignant melanoma, lung metastases, breast metastases, blood–brain barrier, chemotherapy, immunotherapy, radio-immunotherapy, nanoparticle, drug delivery, antineoplastic agents, micelles, hyaluronic acid nanospheres, polymeric nanoparticles, lipid nanoparticles, magnetic nanoparticles, silica nanoparticles, zirconium nanoparticles, radiosensitisers, nanoscale immunoconjugates

## Abstract

(1) Background/Objectives: Primary and secondary brain tumours often hold devastating prognoses and low survival rates despite the application of maximal neurosurgical resection, and state-of-the-art radiotherapy and chemotherapy. One limiting factor in their management is that several antineoplastic agents are unable to cross the blood–brain barrier (BBB) to reach the tumour microenvironment. Nanomedicine could hold the potential to become an effective means of drug delivery to overcome previous hurdles towards effective neuro-oncological treatments. (2) Methods: A scoping review following the PRISMA-ScR guidelines and checklist was conducted using key terms input into PubMed to find articles that reflect emerging trends in the utilisation of nanomedicine in drug delivery for primary and secondary brain tumours. (3) Results: The review highlights various strategies by which different nanoparticles can be exploited to bypass the BBB; we provide a synthesis of the literature on the ongoing contributions to therapeutic protocols based on chemotherapy, immunotherapy, focused ultrasound, radiotherapy/radiosurgery, and radio-immunotherapy. (4) Conclusions: The emerging trends summarised in this scoping review indicate encouraging advantageous properties of nanoparticles as potential effective drug delivery mechanisms; however, there are still nanotoxicity issues that largely remain to be addressed before the translation of these innovations from laboratory to clinical practice.

## 1. Introduction

Conventionally, brain tumours are broadly classified into primary and secondary: the former can originate from any tissue of the central nervous system (CNS), whereas secondary tumours spread into the brain from elsewhere. Whereas primary brain tumours can be either benign or malignant, secondary tumours are by definition cancerous lesions. Amongst the primary tumours of the CNS, gliomas are the most frequent and devastating type [1]. Those tumours can be further classified as per their aggressiveness and extent of proliferation, according to the 2021 World Health Organisation (WHO) document, into grades I and II gliomas (low-grade gliomas (LGGs)) grade III and IV (high-grade gliomas (HGGs)) [2]. In clinical practice, the least malignant form is pilocytic astrocytoma, whereas the most malignant one is glioblastoma (GBM) [2]. While GBM accounts for the most frequent subtype of primary brain tumours, other classes characterised by various degrees of local aggressiveness, such as meningiomas, are the runners-up in terms of incidence and come up on top in terms of prevalence, reflecting the operative volumes of those lesions [3]. On the other hand, secondary brain tumours (also known as metastases) are the most common forms of brain tumours in adults, and their diagnosis is increasing proportionally to the incidence and prevalence of cancer, which has been defined as a silent pandemic by the Cancer Committee of the ACS (American College of Surgeons) (https://www.facs.org/quality-programs/cancer-programs/, accessed on 10 December 2024).

Despite the application of aggressive treatment strategies, the prognosis of brain tumours is dismal. HGGs are among the most lethal of all cancers, with a median overall survival (OS) of 14 to 20 months after optimal multimodal therapy [4]. Unfortunately, even LGGs do not boast encouraging outcomes due to the evolution to anaplasia that characterises the natural history of LGGs, leading to death within 5–10 years [5]. Clinicians struggle to predict individual patients’ outcomes from other primary and secondary brain tumours due to their heterogeneity (clinically and histologically). Nonetheless, some commonalities regarding their anatomical- and treatment-specific factors have been considered by surgeon-scientists to improve the quality of neuro-oncology care offered to those patients.

In the last two decades, the advent of nanomedicine has allowed for a quicker transition of new innovations from the laboratory to clinical wards and operating rooms, a transformation that has had profound implications in neurosurgery [6]. Accordingly, this study aims at showcasing the prospective changes in our diagnostic and therapeutic paradigms for primary and secondary brain tumours. Therefore, an accurate understanding of the mainstay of neurosurgical management for these lesions is propaedeutic to presenting the new strategies brought up by nanomedicine, around which this study is centred.

## 2. Evolution of Treatment Modalities for Primary and Secondary Brain Tumours

The mainstay of neurosurgical treatment for brain tumours focuses on aggressive gross total resection, aiming for >95% tumour resection. An all-or-none approach towards tumour cytoreduction was demonstrated to be particularly important in GBM patients by the MD Anderson Cancer Centre Neurosurgical Group [7]. Their data concluded a statistically significant correlation between survival and >98% tumour volume resection, a correlation that is much weaker in secondary brain tumours, whose survival depends on many other factors related to the staging and response to treatment of the primary lesion.

Following tumour cytoreduction, radiation therapy with concurrent or adjuvant chemotherapy is commenced within 30 days for both HGGs and secondary brain tumours [8], while for brain metastases, the choice of adjuvant chemotherapy is highly variable depending on the WHO class and grade of the primary lesion, in HGGs the first-line treatment consists of the use of temozolomide (TMZ) [9,10] in the context of the Stupp protocol, the gold standard therapy for grade 4 gliomas. In fact, a statistically significant increase in 2-year survival can be achieved with the use of RT plus concomitant and adjuvant TMZ for GBM, from 10.4% to 26.5% according to the largest international randomised clinical trial published by Stupp et al. [10]. Nonetheless, this approach, which was so successful in HGG, does not fully translate as a treatment modality for LGG [11,12]. In their multivariate analysis, Nitta et al. [13] showed the extent of resection (EOR) to be significantly associated with progression-free survival (PFS) and OS; nonetheless, radiotherapy (RT) was not associated with better outcomes. This led to the conclusion that treatment for this class of gliomas should aim for maximal resection and continuous follow-up, with the understanding that aggressive treatment with the use of chemotherapy and RT should be reserved only for tumours carrying poor prognoses like diffuse astrocytoma or those converting to high grades.

Various shortcomings currently limit the efficacy of neuro-oncological treatments in primary and secondary brain tumours. Firstly, it should be noted that various first and secondary lines of chemotherapy are currently available for brain tumours. These include alkylating agents such as lomustine and cisplatin; anthracyclines such as Doxorubicin; topoisomerase inhibitors such as Irinotecan; and plant alkaloids such as Vinblastine. Unfortunately, all these chemotherapy classes pose challenges due to their systemic toxicity, which results from either the chemotherapeutic drugs having poor efficacy in brain penetration or their short half-life. Those aspects oblige neuro-oncologists to administer high pharmacological dosages with consequent multifold side effects (e.g., haematopoietic toxicity, hepatotoxicity, nephrotoxicity, ototoxicity, and pulmonary toxicity). Secondly, given the focus on radical resection in primary and secondary brain tumours, the efforts of the surgical community have been aimed at preserving patients’ executive function postoperatively. The prospective improvement and the often transitory nature of the functional impairments caused by radical surgical interventions were initially evidenced by Talacchi et al. in a large GBM cohort [8]. For this, the push toward radical resection gave rise over time to continuous improvements in preoperative planning aimed at minimising iatrogenic insults in any patient harbouring brain lesions.

### 2.1. Surgical Planning and Prediction Models

For a long time, tumour localisation and the associated radiological characteristics (e.g., midline shift of ≥1 cm, subcortical positioning and insular location) have been considered significant predictors of incomplete tumour removal [8]. However, multiple surgical aids, such as the use of neuro-navigation based on functional magnetic resonance imaging (fMRI) and the use of intraoperative computed tomography (iCT), real-time intraoperative ultrasound (IoUS), and intraoperative neurophysiology (IN), have progressively emerged as game changers [14,15]. These aids for the surgical removal of the tumour have been responsible for the better rates of PFS, OS, and functionally independent survival obtained in recent years. Nonetheless, case complexity greatly influences outcomes in neurosurgery; hence, grading scales that quantify brain damage and introduce valid surgical efficacy indicators have been proposed [16]. Such tools inform clinicians about the chances of achieving radical EOR and the risk of postoperative complications; nonetheless, they are subject to ongoing refinement meant to address their shortcomings [17]. For instance, the capability to preoperatively quantify the risk of iatrogenic brain damage is outshone by the progressive improvement in our imaging modalities and surgical aids. While the grading scale proposed by Marcus et al. [16] used conventional magnetic resonance imaging (MRI) to formulate predictions, Saraswathy et al. [18] demonstrated that marginal gains in surgical planning could be offered by advanced MRI sequences, such as diffusion-weighted MRI and proton MR spectroscopic imaging (^1^HMRSI). This underscores the contribution of biomedical engineering to neuroimaging, but nanotechnology has also been demonstrated to be a potential actor for positive change. In fact, the development of high-performance contrast agents based on nanocomposites has recently received considerable attention because these new agents hold great promise and potential for more effective and safer cancer diagnosis and intraoperative visualisation of the tumour, including the presence of possible foci of disease residuals once the intended resection is completed [19]. Additionally, other prediction models, such as the one proposed by Marko et al. [20], allowed plotting the relationship between survival probability and adjuvant therapy received by the patients, putting paramount importance not only on preoperative planning and surgical management but also on postoperative treatments. This conclusion underscores the attention of the neuro-oncology community towards innovative pharmacological strategies, including those enabled by nanotechnologies, and is consistent with the grand objective of our scoping review.

### 2.2. Current Strategies for Radiation Therapy

While conventional RT following tumour debulking remains the preferred choice for GBM and uncontrollable metastatic disease to the CNS, clinicians have been advocating for a more tailored radiation therapy for brain tumours suitable for adults and paediatric cases, with the goal of reducing side effects and improving quality of life [21,22]. Stereotactic radiosurgery (SRS) is defined as a technique of closed skull destruction of a predetermined intracranial target by a single-fraction, high dose of ionising radiation using a precision stereotactic apparatus [23]; this technique boasts the benefits of minimising collateral cells’ exposure to irradiation and delivers, with greater accuracy, high ablative doses centrally to the target margin [24]. SRS comes in many different forms, depending on the type of penetrating radiation utilised, Gamma Knife, Linear Accelerators, etc. [24,25,26]. These techniques can prove to be quite advantageous for small tumours (<3 cm^3^), either alone or in combination with other surgical and endovascular treatments. In fact, SRS can also be used as a primary treatment for various primary tumours, such as meningiomas in which a 5-year tumour control rate of 85–100% has been demonstrated [27,28,29], as well as brain metastases. That said, even SRS is not a panacea: Gong et al. [29] highlighted that SRS using a single-fraction Gamma Knife still has limited use in tumours located close to critical intracranial anatomical structures (e.g., optic nerve, pituitary stalk, etc.) due to their radiation tolerance. To counteract this problem, scientists have investigated radiation and pharmacological strategies meant to effectively reach the CNS, protect the healthy cells in critical anatomical structures surrounding the tumour target, and prevent direct damage following ablative SRS, including long-term consequences, such as malignant transformation over time [30]. Pharmacological agents which increase the toxic effects of radiation therapy are called radiosensitisers and radioenhancers (agents which reduce the total amount of radiation required to be lethal to a given population of tumour cells) [31,32,33]; however, access to the CNS represents a specific challenge for any neuro-oncological treatments, and nanosolutions have been advocated for to address this specific challenge.

### 2.3. Resistance of Tumour Cells to Chemotherapy

As mentioned above, whilst chemotherapy is often indicated in the treatment of brain tumours, there are significant challenges posed by CNS penetration. However, it has also been demonstrated that over time, tumour cells develop a resistance to these chemotherapeutic agents [34,35,36]. One of the proposed mechanisms for this is through tumour cells’ intrinsic DNA mismatch repair mechanisms and the upregulation of specific drug-resistant proteins following long-term exposure to the chemotherapeutic agent. For example, it has been described that the overexpression of the O-methylguanine-DNA methyltransferase (MGMT) protein in glioma cells leads to the inactivation of TMZ through omission of the alkyl or methyl group which is vital to its mechanism of action [34]. Similar mechanisms are in play in metastatic tumours as well. As an example, Lee et al., describe how multiple myeloma cells that are sensitised to CD40 demonstrate a marked increase in the expression of the multi-drug-resistant protein 1 (MRP1) via the AKT signalling pathway [36]. This protein then contributes to chemoresistance to Vincristine by limiting cellular uptake of the drug. The utilisation of nanoparticles for drug delivery can significantly improve drug penetration by shielding the active pharmacological substance, thus increasing its therapeutic concentrations in tumour cells and, as such, combat drug resistance, as will be further discussed.

## 3. Emerging Treatment Modalities Based on Nanomedicine

### 3.1. Overcoming the Blood–Brain Barrier Using Nanoparticles

The selectivity of the blood–brain barrier (BBB) and the blood–tumour barrier (BTB) has emerged as an important reason behind the poor effectiveness and outcomes of antineoplastic agents. The BBB is a tight barrier, formed primarily through brain capillary endothelial cells, as well as a basement membrane, that protects the brain and only allows the crossing of essential substances, such as glucose and amino acids [37,38]. Proteins such as transferrin and Lactoferrin can only cross this barrier via receptor-mediated endocytosis [37], and the passive passage through BBB is only possible for lipophilic drugs, which carry a molecular weight of less than 400 Da and eight hydrogen bonds [35]. The BTB is comprised of abnormal vessels that enclose the tumour cells and increase the interstitial pressure within the tumour microenvironment. As an estimate, 98% of small molecules and 100% of large molecules fail to achieve therapeutic levels due to a failure to sufficiently reach the brain [39], largely due to the obstacle of the BBB. Nanoparticles (NPs), on the other hand, are able to encapsulate these molecules and provide specific transportation across the BBB via specific ligands attached to their surface. These can bind to key receptors present at the BBB, hence providing the possibility of tackling previously unreachable tumours like GBMs [40]. This strategy has been proposed in various forms for enhanced preoperative and intraoperative imaging, more effective chemotherapy protocols, and safer radiation treatments [41].

### 3.2. Towards Nanosolutions

Nanomedicine aims at using nanostructures, possibly with biodegradable characteristics, to find new solutions to old problems: for instance, hyaluronic acid (HA) nanospheres have been suggested as BBB/BTB carriers. Attention was drawn towards HA for its immunoneutral, biocompatible, and biodegradable properties [42], which enable NPs containing HA (HA-NPs) to easily bypass the BBB due to the action of reception-mediated endocytosis, hence improving the performance of chemotherapeutics and contrast agents [42]. HA-NPs can also exert specific tumour-targeting activity, which is due to the interaction that occurs between hyaluronidases found in the extracellular matrix (ECM) and HA receptors located on the bilipid membrane of tumour cells [43]. Furthermore, primary brain tumours induce the remodelling of the ECM, and HA, which is one of its key components, has been demonstrated to increase fourfold (to levels comparable to those seen in CNS development) in primary brain tumours [42,44]. For all those reasons, Jeong et al. [45] proposed 100–200 nm HA-NPs conjugated with cisplatin to target glioma tumour cells lines. As such, they were the first research group who successfully observed an increased cisplatin release when those NPs were tested in a glioma cell line (U343MG) which releases hyaluronidases. Their proof of concept triggered further studies to test whether HA-NPs could serve as suitable antitumour carriers and delivery systems even in secondary brain tumours. To assess that, HA was successfully used to transport cisplatin, a potent chemotherapeutic and radiosensitiser [46].

The overexpression of receptors such as CD44 and RHAMM in brain tumours was another reason to consider HA-NPs. Since CD44 is a transmembrane glycoprotein and a primary cell surface receptor for HA, hyaluronic acid–ceramide (HACE)-based nanoprobes were utilised for MRI and demonstrated raised uptake of these nanoprobes in cancer cell lines with high CD44 receptor expression [47]. This showed that MRI contrast agents have greater tumour targetability when the strong affinity of HA and CD44 is exploited, suggesting that advantageous properties of HA-NPs can go beyond drug delivery systems to enhance neuroimaging protocols [47]. HA oligomers (o-HA) have also proven to be advantageous in the sense that they antagonise the malignant properties of glioma cells by competing for the endogenous HA polymeric interactions, which, as a result, interrupts HA-induced signalling [48].

This background on the applications of nanomedicine to address many unmet neurosurgical needs in the management of primary and secondary brain tumours justifies our interest in this niche of neuro-oncology. The information provided in this introductory section warrants a deeper appraisal of the scientific literature to understand how successful the harnessing of nanomedicine has been in tackling the specifics of brain tumours’ microenvironment [49] and delivering innovative antineoplastic agents and immunotherapeutic, radiotherapeutic, and anti-angiogenic drugs to the CNS. Given the exploratory nature of our research quest, a scoping review represented the best way to progress forward.

## 4. Materials and Methods

### Scoping Review Methodology

This scoping review hopes to explore recent developments in nanomedicine and its capability of delivering antineoplastic agents for the treatment of primary and secondary brain tumours. Nanomedicine as a means of drug delivery poses several advantages such as its ability to deliver poorly water-soluble drugs, its targeted drug delivery, and its transportation of large macromolecules to intracellular sites. The pharmacological targets can also be visualised in real time by integrating imaging modalities and harnessing an optically modulated delivery of therapeutic agents [50].

This scoping review was conducted in the summer of 2024 according to the PRISMA-ScR guidelines and aims to (1) identify hot topics and emerging trends in the utilisation of nanomedicine in drug delivery of primary and secondary brain tumours and (2) demonstrate whether and how nanomedicine is extending survival and quality of life in patients diagnosed with primary or secondary brain tumours.

**Figure 1:**
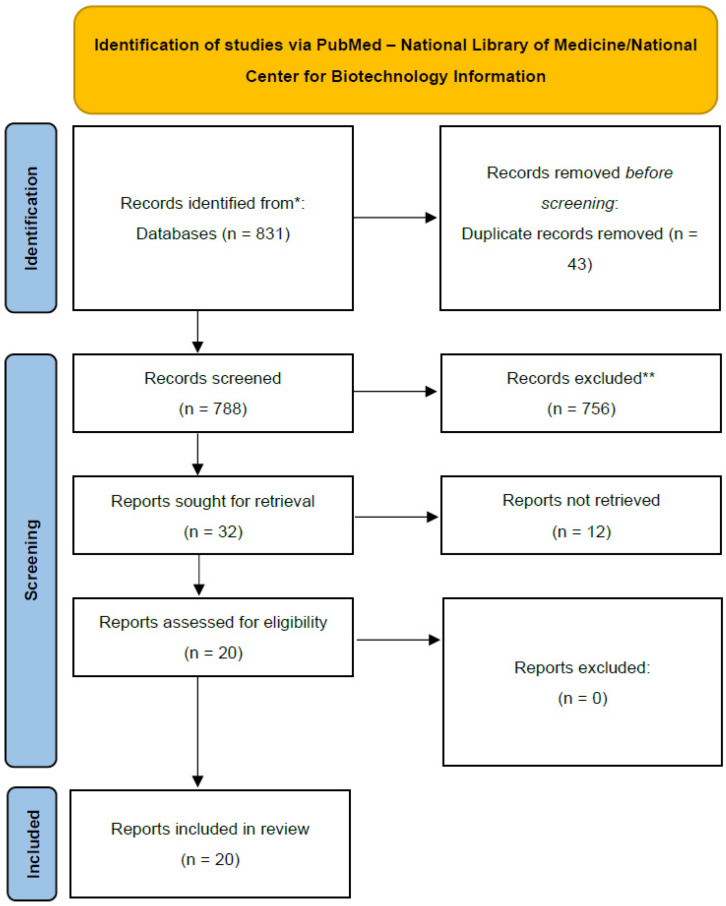
Flow diagram according to PRISMA-ScR guidelines, (https://www.prisma-statement.org/scoping/, accessed on 23 June 2024); * indicates that database used for the search (https://pubmed.ncbi.nlm.nih.gov/, accessed between 23 June 2024 and 31 October 2024); ** indicates all articles excluded at the time of abstract review due to either their focus (e.g., articles on the management of brain tumours not dealing with the use of nanoconjugates or articles on nanomedicine not focused or not fully dedicated to neuro-oncology) or design (non-original investigations such as case reports, editorials, and letters to editors).

An array of search terms was input into PubMed—National Library of Medicine/National Center for Biotechnology Information, with the time range being set between 2010 and 2024, to find relevant articles for triaging and inclusion in this review. No language restrictions were placed on the initial search. To maximise the chances of identifying relevant trends in the management of secondary brain tumours, the most common histotypes (breast and lung cancers) and the tumour whose oncological protocols have changed the most in recent years (melanoma) were considered [51]. The following MeSH terms, and combinations of them, were therefore used: “Nanomedicine + glioblastoma + drug delivery”; “Nanomedicine + blood brain barrier + drug delivery + brain tumour”; “Nanomedicine + brain metastases + drug delivery”. A flow diagram reflective of the various steps undertaken in this scoping review is presented in Figure 1.

All key statements made in this scoping review have been appropriately referenced, and a comprehensive numerical list can be found in the References section.

The Results section (Section 5) lays out all the appropriate articles that were retained for analysis in this scoping review following our initial literature search. Conflicts of opinion regarding the inclusion of any given article in this review were resolved among the authors by discussing the pros and cons through a conventional Delphi methodology (of note, this led to the exclusion of 12 articles which were not deemed relevant enough to be listed in the summative tables of this scoping review).

Data have been collected, analysed and presented in a systematic format within the main text of Section 5, as well as in the form of two summary tables presented at the end of each major subsection of the following section, where a synthesis of results is provided. Final reporting has been drafted and verified before submission against the PRISMA-ScR checklist (https://www.prisma-statement.org/scoping/, accessed on 23 June 2024).

## 5. Results

The highest number of articles (n: 657) was found when the search term “Nanomedicine + brain tumour+ chemotherapy + drug delivery” was input. Next, the search terms “Nanomedicine + Immunotherapy + brain tumour + drug delivery” and “Brain tumour metastasis + Breast + Nanoparticle” generated 51 and 71 results, respectively. The terms “Nanomedicine + brain tumour+ antiangiogenic therapy + drug delivery”; “Brain tumour metastasis + melanoma + nanoparticle” and “Brain tumour metastasis + lung + nanoparticle” generated between 8 and 25 results. Lastly, the term “Nanomedicine + meningioma + drug delivery” only generated one result.

This scoping review highlighted various strategies exploited by different NPs to bypass the BBB and contribute to therapeutic protocols based on chemotherapy, immunotherapy, focused ultrasound, RT/SRS, and radio-immunotherapy. Those findings will be presented in the following subsections, which will cover four main areas: NPs and their various theranostics use [52,53,54,55,56,57,58,59,60,61,62,63,64,65,66,67,68,69,70,71,72,73,74], immunotherapy [75,76,77,78,79,80,81,82,83,84,85,86,87,88,89,90,91,92,93,94,95,96,97,98,99,100,101,102,103,104,105,106,107,108,109,110,111,112,113,114,115,116,117,118,119,120,121,122,123,124,125,126,127,128,129,130,131,132,133,134,135,136,137,138,139,140], radio-immunotherapy [141,142,143,144,145,146,147,148,149,150,151], and anti-angiogenic therapies [152,153]. The 20 studies retained after completion of the screening process are presented in the subsection below and summarised in Table 1 and Table 2.

### 5.1. The Blood–Brain Barrier and Chemotherapeutic Drug Delivery via NPs

As mentioned in Section 3.1, the BBB can greatly limit the efficacy of antineoplastic therapeutic agents since it actively removes these agents with the means of efflux transporters like P-glycoprotein (P-gp). Paracellular diffusion is also prevented by means of tight junctions between endothelial cells [52,53]. NPs can be designed to solve this issue by encapsulating numerous drugs, bypassing the BBB and BTB, and minimising the off-target effects on the surrounding healthy tissues [52]. Targeted brain tumour nanodrug delivery can be achieved by the encapsulation of multiple pharmacological agents and by the exploitation of multiple different signalling pathways all at once. In this subsection, a dual approach will be used to summarise the evidence from the literature. On one hand, the most relevant types of NPs will be presented; on the other hand, various modalities where those NPs are used to grant passage into the CNS and tackle brain tumours will also be described. This dual approach will allow us to comprehensively cover this relevant area of nanomedicine, from strategies to increase BBB permeability, photodynamic approaches and thermotherapy, and from ultrasound-modulated chemotherapy to the use of radiosensitisers in various forms of radiation therapy.

#### 5.1.1. Polymeric NPs

Polymeric conjugates are composed of soluble polymeric NPs that are loaded with antineoplastic agents to aid site-specific selectivity and evade processes that inhibit the efficacy of delivery of these antineoplastic agents such as protein-induced immunogenicity. In addition, these polymeric NPs can encapsulate hydrophobic drugs and increase their bioavailability.

Chang et al. [54] conjugated cisplatin with Pluronic F127-complexed PEGylated poly(glutamic acid) to produce an NP called PLG-PEG/PF127-CDDP. The NP was used on GL261 glioma cells, and a 72.53% cell invasion reduction was seen in in vitro studies. The circulating half-life of cisplatin was also increased to 9.75 h in vivo, which caused, by day 16 post-treatment, a tumour size reduction by 50%.

Annonaceous acetogenins (ACGs), a family of naturally occurring polyketides isolated from various species of the plant family Annonaceae, have been shown to have potent anti-tumour activity [55]; for instance, the monomeric component of ACG called bullatacin exhibits therapeutic activity that is 300 times that of Paclitaxel for leukaemia [56,57]. However, the delivery of ACGs is challenging due to their form being a viscous solid, making dissolution into water difficult. Ao et al. [57] used the amphiphilic polymer Poly(ethylene oxide)-b-poly(butylene oxide) (PEO-PBO) to deliver ACGs in vivo whilst also evading mononuclear phagocyte-driven NP clearance. PEO-PBO carried nanomicelles loaded with ACGs called ACGs/EB-NCs in the following three forms: ACGs/EB_5_-NCs, ACGs/EB_10_-NCs, and ACGs/EB_20_-NCs (depending on the ACGs/EB-NC feeding ratios). Compared to the release of free ACGs, the cumulative release rates of these three forms of ACGs/EB-NCs were significantly stronger in U87 MG cells, with ACGs/EB_5_-NCs showing the highest release rate of 78.2% (within 216 h). Interestingly, though the nanomicelle ACGs/EB_20_-NCs had the smallest cumulative release rate of 56.3% within 216 h, it showed the smallest half-inhibitory concentration and largest tumour inhibition rate.

**Figure 2:**
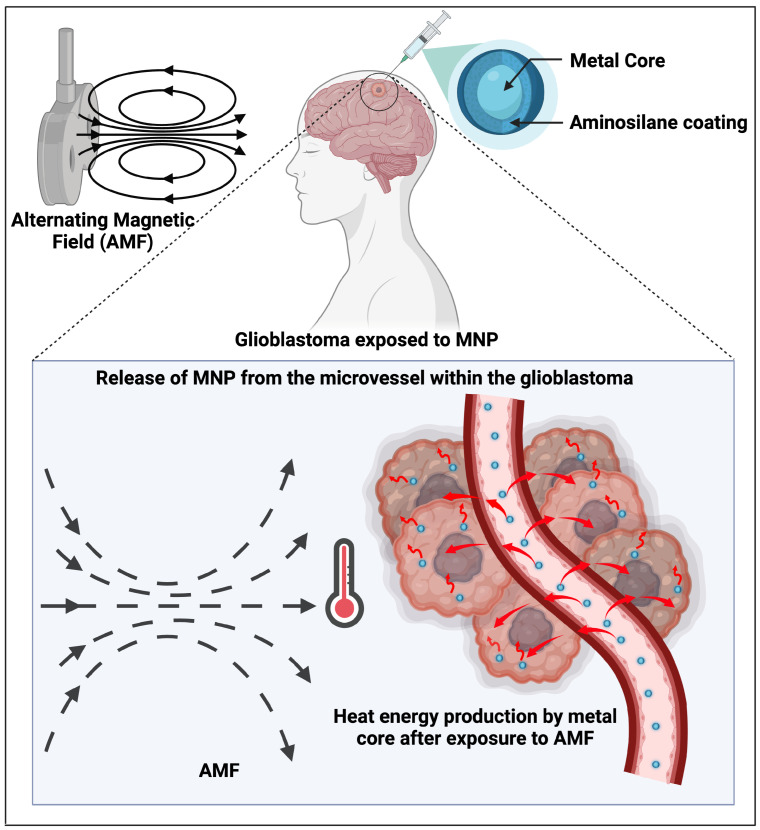
Dual targeting of primary malignancy and brain metastases. This image shows how polymeric nanoparticles loaded with Platin-M and chemotherapeutic glycolytic inhibitors are able to enter the mitochondria of primary malignancy in breast or lung cancers, as well as their secondary brain lesions. This approach has been proposed in breast-induced brain metastases by Ashokan et al. [70]. Created in BioRender. Khilar, S., 2025 (https://BioRender.com/d46l747/, last modified on 21 January 2025).

Finally, polymeric NPs have been used for dual action on brain metastases and their primary tumours. For instance, Ashokan et al. [70] developed polymeric NPs loaded with a combination of Platin-M, a pro-drug of cisplatin, and a glycolytic inhibitor called mitochondrion-targeted dichloroacetate (DCA). In their study, the engineered nanocarrier had a terminal triphenylphosphonium (TPP) cation that could link the hyperpolarised membrane of mitochondria, which are known to be involved in ageing and carcinogenesis [112,113]. The ability to penetrate the BBB as well as the mitochondrial hyperpolarised membrane allowed the simultaneous targeting of both cancer cells located at the primary peripheral organ site, as well as those within the CNS (see Figure 2).

#### 5.1.2. Lipid NPs

Liposomes and niosomes are two examples of self-assembling concentric vesicles, which have the ability to encapsulate molecules of water-soluble, lipid-soluble, and amphiphilic nature [72]. Liposomes can also be PEGylated by the addition of polyethylene glycol chains, which can increase their half-life [72]. Further examples of lipid NPs include solid lipid NPs (SLNs), in which drugs are inserted into a lipid nucleus or core. The lipophilic nature of lipid NPs makes them a good candidate for passing through the BBB via passive diffusion or transcytosis, either receptor-mediated or adsorptive-mediated [73]. Moreover, Medes et al. describe in their in vitro study how ultra-small nanostructure lipid carriers (usNLCs) can be coupled with specific cell-penetrating peptides (CPPs), tumour-targeting peptides (TTPs), stearylamine, or transferrin, to enhance their permeability across the BBB as well as uptake into glioma cells for more targeted drug delivery [73]. Additionally, Joshy et al. demonstrated the successful uptake of zidovudine to glioma cells using modified SLNs in their in vitro study [74].

#### 5.1.3. Magnetic NPs

**Figure 3:**
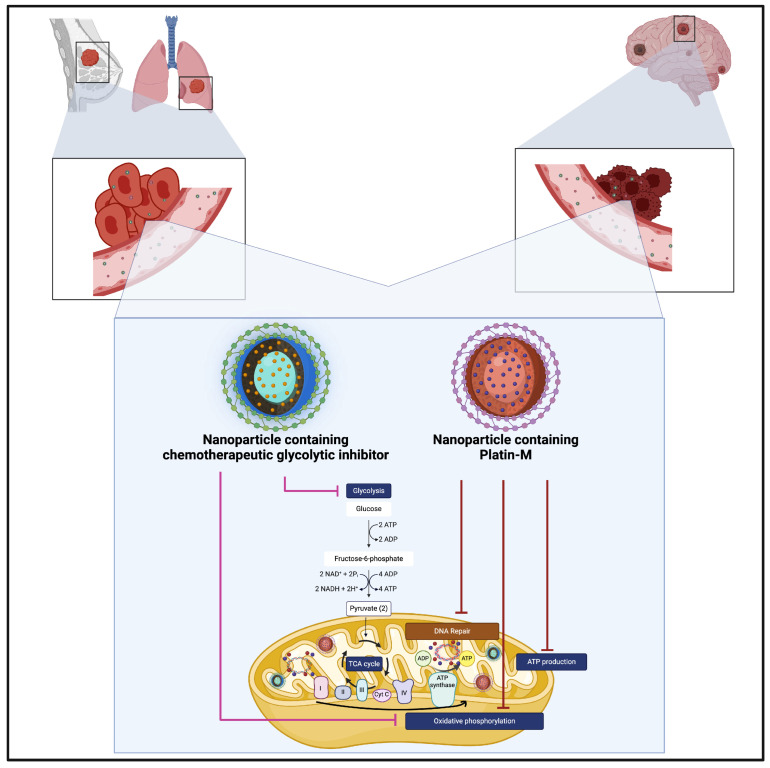
Intratumoural thermotherapy can be achieved by directing an alternating magnetic field towards nanoparticles containing a magnetic core. This strategy has been adopted in patients with recurrent glioblastoma by Maier-Hauff et al. [60]. Created in BioRender. Khilar, S., 2025 (https://BioRender.com/c14m563/, last modified on 21 January 2025).

The technique of enhanced permeability and retention (EPR) [58] encompasses the dilation of curved capillaries to reduce blood flow, allowing NPs to permeate through the 20–200 nm wide pores of vessels and subsequently accumulate within the tumour—these are typically sized 3–200 nm in diameter [59]. Magnetic NPs (MNPs) are advantageous due to their response to the external magnetic field (EMF) and are used for theranostic purposes (see Figure 3). Therapeutic agents can be conjugated with MNPs, and, via magnetic targeting, these MNPs can be vehiculated to the tumour site where, their local concentration can be increased to remarkably improve their therapeutic efficacy. As such, when these MNPs accumulate, their exposure to EMF causes cell destruction via heat generation [59].

A notable study by Maier-Hauff et al. [60] applied thermotherapy using an alternating magnetic field (AMF) and injecting directly into the tumour a magnetic fluid containing supermagnetic NPs, with an iron concentration of 112 mg/mL, aqueously dispersed, yielding superior responses in comparison to the standard of care [61,62].

#### 5.1.4. Combining MNPs with the Technique of Focused Ultrasound to Increase BBB Permeability and Drug Delivery via NPs

Liu et al. [63] combined the properties of focused ultrasound (FUS) and MNPs to enhance the delivery of chemotherapeutic agents across the BBB to the tumour site while allowing for MRI monitoring during treatment. FUS reversibly disrupts the BBB and increases its permeability with the use of microbubbles and a low-energy burst tone. Due to its effects being local rather than systemic, off-target side effects are reduced. The two techniques negate the less efficient passive diffusion technique to cross the barrier due to the presence of magnetic targeting and increase the concentration of MNPs within the tumour site. The aqueous solution of the MNPs can be stabilised, for instance, by encapsulating iron oxide (Fe_3_O_4_) within poly [aniline-co-N-(1-one-butyric acid)] aniline (SPAnH) as a surface layer. Then, cytotoxic anti-cancer agents can be immobilised on the surface of MNPs to reduce the therapeutic quantity required. In the study conducted by Liu et al., control animals that were treated with Epirubicin-MNP without FUS had no MNP accumulation, whereas those treated with FUS/MNPs showed an estimated 15-fold higher therapeutic range of the index drug, Epirubicin, delivered to the tumour site compared to the conventional in vivo administration of a control drug, Doxorubicin (DOX). A decline in tumour volume increase was also seen; the control group had a 313% rise in tumour volume compared to the FUS/MNP group, which saw a 106 ± 24% increase. Additionally, the median survival of the group of interest was 30.5 days compared to the control group, which had a statistically significantly lower median survival of 18.3 days (*p* value = 0.0002).

#### 5.1.5. Silica NPs

Although TMZ’s lipophilicity and small molecular weight allow it to be absorbed orally, its BBB penetration and bioavailability in patients with GBM, or any other brain malignancies where TMZ is one of the only chemotherapy options (e.g., solitary fibrous tumours) are incredibly low at only 20% [10,62,64,65,66]. Additionally, TMZ has a short half-life of 2 h, and efflux pumps present within the brain tumour and BBB cause it not to accumulate sufficiently to cause therapeutic effects. This not only causes less-than-desirable outcomes, such as a 95% likelihood of GBM recurrence within 7 months of diagnosis and a <5% 5-year survival rate, but also causes drug resistance. Numerous types of NPs have been tested to optimize these shortcomings. Mesoporous silica NPs (MSNs) have been shown to exhibit properties such as a large surface area of >1000 m^2^g^−1^, excellent mechanical stability, and drug release, which can be tuned according to internal stimuli such as pH, and external stimuli, such as heat, light, and mechanical field [67,68]. This makes them great NPs for CNS-specific drug delivery systems [66]. Janjua et al. [66] developed novel ultra-small (30 nm) silica NPs with large pores (7 nm) (USLP) as a medium for chemotherapy delivery. This was combined with the Lactoferrin ligand to accommodate BBB crossing and allowed for an increased TMZ accumulation, decreased efflux ratio and improvement in the anti-cancer response [75]. Following those additional tests, Janjua et al. [66] also showed that the efflux ratio of TMZ conjugated with USLP and PEG was significantly lower than that recorded for pure TMZ (0.72 ± 0.11 vs. 2.15 ± 0.18). They also showed that when Lactoferrin as a ligand is coupled with NPs in PEG solution, it accelerates their accumulation within the brain, peaking at about 1 h post administration compared to NPs in PEG solution alone, which peak about 4 h post intravenous administration. This was estimated to be associated with the large amounts of Lactoferrin receptors expressed on the BBB.

#### 5.1.6. Ultrasound-Modulated Chemotherapy: The Case of Zirconium NPs

Wan et al. [69] combined the beneficial properties of NPs formulated in a Zirconium-based framework (UiO-66-NH_2_ NP) with ultrasound to increase the efficiency of TMZ delivery for GBM. Due to internal circulation stability, high loading capacity, and excellent biocompatibility, nanoscale metal-organic frameworks (MOFs), such as UiO-66-NH_2_, provide an unprecedented opportunity for the treatment of cancer, making them an ideal drug delivery vehicle due to the superior cavity volume to load drugs such as TMZ and slow-release functions, which is expected to increase penetration through the BBB. TMZ can in fact be released through the microporous network of UiO-66-NH_2_, and ultrasound accelerated this process via low-frequency oscillations. Wan et al. showed that those Zirconium-based NPs have a loading capacity of 0.25 mg of TMZ per mg of UiO-66-NH_2_. Their study reflected good delivery and enrichment of the TMZ-carrying NPs locally; however, those MOFs loaded with TMZ were not able without ultrasound to inhibit tumour cell migration. The authors explained two possible mechanisms for this dismal result: either the loaded drug’s release is facilitated by the destruction of the carrier, or ultrasound induces changes in the structure of the cells so that the therapeutic agent can reach the local tumour in a targeted manner.

#### 5.1.7. NPs as Radiosensitisers

A Phase I trial called the NANO-RAD [71] trial was conducted on patients with brain metastases who were unsuitable to receive SRS. A novel gadolinium-based 5 nm NP called Activation by Guidance of Irradiation by X-ray (AGuIX) was used with RT on a total of 15 patients with 354 metastases from melanoma, lung, breast, melanoma, and colon cancer. Enhancement on MRI revealed AGuIX to have been distributed across all brain metastases. The median OS and PFS were 5.5 months. Survival 12 months after the end of the study was seen in five patients. AGuIX was also observed to be retained within the tumour for up to one week, supporting its use as a radiosensitiser and potential to be studied in Phase II trials.

### 5.2. Immunotherapy

Although several clinical trials have been conducted and others are still ongoing to test the impact of immunotherapy in the treatment of patients with primary and secondary brain tumours [75], one point that clearly emerged in all these investigations consists of the limitations of new antibody-based drugs to bypass BBB due to their molecular weight and charge. Immunotherapies exploit antigen–antibody interactions and are meant to trigger an immune response against tumours. Peptides (amino acid chains), polysaccharides (chains of simple sugars), lipids, or nucleic acids displayed over the cell membrane of tumour cells can be targeted to tackle tumour growth. In such scenarios, antibodies and aptamers, consisting of short single-stranded DNA or RNA oligonucleotides, are used to target cancer-specific molecules with high affinity in a three-dimensional shape and unchain immune reaction against primary and secondary brain tumours [140]. Given the above, in this subsection, we will cover the use of nanoscale immunoconjugates, immune checkpoints blockade, multiplexing targeting, and use of SiRNA to modulate immunotherapy for brain tumours.

#### 5.2.1. Nanoscale Immunoconjugates (NICs)

The immune system is extremely complex and requires fine-tuning to ensure the protection of the human body. Immune checkpoints are proteins meant to keep our immune system in check, hence avoiding episodes of autoimmunity; nonetheless, they may also prevent an effective response against cancer cells, for instance, by stopping T cells from killing tumour cells in the body [84]. Regulatory T-cells (Tregs) are suppressed, and cytotoxic T-lymphocytes (CTLs), which enact an anti-tumour immune response, are activated with the use of humanised monoclonal antibodies (mAbs). Key examples of these mAbs directed against immune checkpoints are Ipilimumab, Pembrolizumab and Nivolumab, where the Ipilimumab targets the cytotoxic T-lymphocyte-associated antigens (CTLA1-4), whereas the other two drugs target the programmed cell death-1 (PD-1) pathway [76,77]. The interaction between PD-1 and its two ligands (PD-L1 and PD-L2) causes a reduction in the effector T-cell activity by inhibiting the T-cell activation via the kinase-signalling pathway, leading to immunosuppression [85,86]. Checkpoint blockade immunotherapy using antibodies against PD-1 and PD-L1 can block immunosuppressive pathways regulating the T-cells, leading to the enhancement of antitumour immune responses [63]. Hence, neuro-oncologists have tried to replicate the clinical successes obtained when antibodies targeted against PD-1 or its ligands were used to treat immunogenic tumours such as melanomas, renal cell carcinomas, bladder cancer, Hodgkin’s lymphoma, and non-small-cell lung cancer [87,88,89,90,91,92]. The current body of evidence shows an in vitro lack of efficiency by these mAbs when they are administered systemically in glioma murine models [78,79]. Similarly to the strategy adopted for most NPs discussed above, to counteract the issue of antibodies not being able to cross the BBB, Galstyan et al. [80] designed an NIC, where antibodies against CTLA-4 and PD-1 were covalently bonded to a drug carrier called the poly (Beta-L-malic acid) PMLA backbone. These NICs cross the BBB and reach brain tumours using transcytosis mediated by transferrin receptors (see also Section 5.2.3). When antibodies against CTLA-4 and PF-1 were administered intravenously, after 4 and 6 h, they were barely detectable outside of blood vessels. In contrast, when they were delivered via NICs, they were detectable within the tumour parenchyma only (but not elsewhere in the brain) within 4 h. This led to the deduction that NICs hold the ability to cross the BBB and selectively accumulate within brain tumours. The survival of mice with GBMs that were treated with these NICs containing a combination of antibodies against CTLA-4 and PD-1 was significantly longer compared to when free antibodies targeted against these immune checkpoint inhibitors were administered or if only a single checkpoint inhibitor was targeted.

#### 5.2.2. Co-Encapsulating Paclitaxel with Immune Checkpoint Inhibitors

Zhang et al. [81] also looked at antibodies against PD-1, but the innovative approach in their study was that they loaded those antibodies against PD-1 (aPD-L1) into redox-responsive micelles and combined them with Paclitaxel (PTX), a chemotherapeutic drug. The combination of antibody and the chemotherapeutic agent in a nano-micelle with angio-pep2 (A2) peptide was termed A2 APM by the investigators.

T-lymphocyte activation, the production of damage-associated molecular patterns (DAMPs) by dying cells, and dendritic cell maturation are phenomena that have been linked with immune cell death due to chemotherapy [82,83]. The point of interest here is the activation of T-lymphocytes. This combination allowed the micelles to penetrate the BTB: using an in vitro BTB model, the study showed that the utilisation of A2 peptide allowed aPD-L1 nano-micelles to cross the cell monolayers. They also showed a greater half-life for A2APM compared to free aPD-L1 (33.05 h and 23.86 h, respectively), which was noted to be associated with decreased aPD-L1 clearance as they were in a PEG shell.

Accumulation testing was carried out via aPD-L1 labelling with Cy7.5 dye. After A2APM, free aPD-L1 and APM samples were intravenously administered, and it was seen that A2APM accumulated in greater amounts 72 h post administration compared to the other groups with statistical significance. The A2APM combination also showed significant tumour regression after day 7 and 60% of A2APM mice bearing the GL261 tumour line had a striking reduction in their tumour size, exhibiting superior tumour regression properties in comparison to mice, which were treated with free-PTX, A2AM, and A2PM. The authors suggested that those latter groups were not able to elicit a strong tumour regression response due to inadequate brain accumulation.

The improved survival of the A2APM group, in comparison to free aPD-L1 and free PTX, was further explored post-resection of the gliomas: mice injected with A2APM after having their GBM tumours surgically removed under a microscope did not show any infiltrating tumour cells around the resection site or elsewhere in the rest of the normal brain parenchyma, whereas mice who received free aPD-L1, free PTX, and APM all had infiltration by tumour cells within the brain.

#### 5.2.3. Immunotherapy with Multiplexing Targeting

The development of brain metastases can affect between 8 and 10% of adults with cancer [93]. Primary tumours from the breast and lung and melanoma contribute to the formation of most of these brain metastases [51,94]. Brain metastases from breast cancer are often diagnosed late, as only when the disease burden is significant do neurological symptoms manifest [95,154].

Human epidermal growth factor receptor (HER) overexpression is a biological signature of breast cancer, as this oncogenic receptor is also implicated with apoptosis avoidance and drug resistance via the coupling with the PI3K/Akt cell-signalling pathway [96,97,98,99]. Lim et al. [100] loaded hyperbranched polymers (HBPs) with DOX, and, to achieve tumour targeting, labelled the NP with anti-HER3/anti-PEG bispecific antibody fragments. This served as one of the first attempts to use nanotechnology to refine immunotherapy strategies for brain metastases. However, the treatment of brain metastases secondary to breast cancer can be challenging despite the use of Trastuzumab (TZ), an anti-HER2 antibody that has been shown to ameliorate patients’ survival; in fact, TZ has poor penetrance to the CNS [101,102,103].

**Figure 4:**
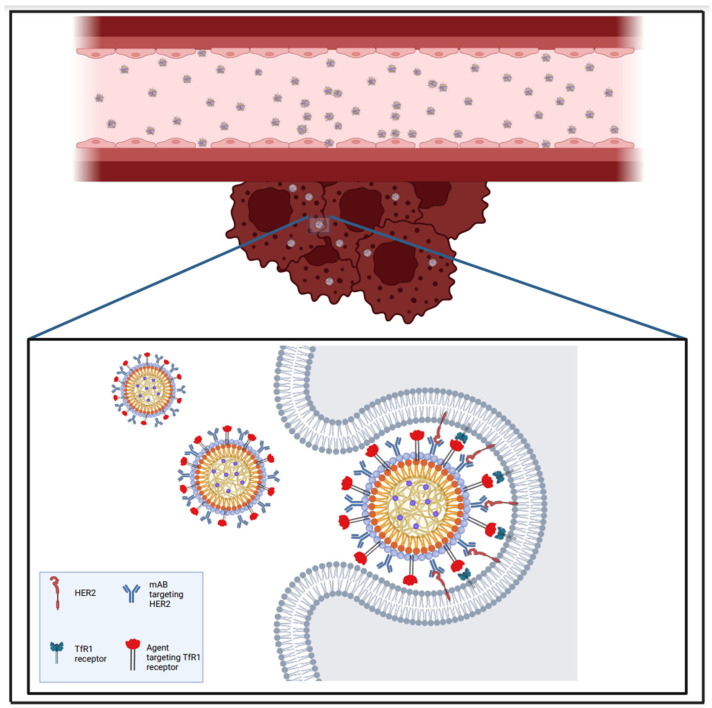
Immunotherapy can exploit targeted drug delivery to induce a tumour-hostile microenvironment. This approach has been used by Sevieri et al. [107] to create ferritin nanoparticles able to target HER2 and TfR1 receptors on the surface of tumour cell membranes and penetrate those cancer cells via receptor-mediated transcytosis. Created in BioRender. Khilar, S., 2025 (https://BioRender.com/c82g636/, last modified on 21 January 2025).

As seen in previous paragraphs, NPs have been used to bypass BBB, and with regard to metastatic lesions, one of the proposed strategies revolved around ferritin NPs (HFn) binding to the transferrin receptor 1 (TfR1) [104,105,106]. Sevieri et al. [107] conjugated TZ and HFn to compose an NP, termed H-TZ, which could specifically target HER2, as well as the TfR1 (see Figure 4).

When these H-TZ NPs were combined with Docetaxel (H-TZ + Dtx) in murine models, a statistically significant reduction in tumour growth was observed 7 days after tumour implantation in the treatment group compared to the mice group treated only by Docetaxel. Compared to free TZ, the accumulation of H-TZ within the tumour was significantly greater as per their immunofluorescence signal intensity. Impressively, H-TZ + Dtx also showed more uniform distribution on the membrane of the cancer cells compared to free TZ, further confirming that it has properties of accurately targeting the HER2+ tumour cells. It was also found that in mice treated with H-TZ + Dtx, a significant reduction in tumour development was linked to macrophage activation around tumours, suggesting that targeted TZ accumulation helped shape an anti-tumoural microenvironment. Such mechanisms are likely driven by macrophage activation post interaction between their receptors and the antibodies bound to cells, which then trigger cancer cell killing in an antibody-dependent fashion [108,109].

#### 5.2.4. Immunotherapy Plus siRNA

Sevieri et al. [107] already showed the effective inhibition of tumour growth when a combination of TZ and Docetaxel is used and delivered via NPs. However, Ngamcherdtrakul et al., 2022 [110], combined these two agents with a siRNA against HER2 using a hydrodynamic 100 nm NP. When tested on the HER2 + HCC1954 drug-resistant tumour mouse cell line, significant tumour growth inhibition was seen when compared to the delivery of free Docetaxel. Similarly, tumour growth inhibition was seen when tested on HER2 + BT474 tumours in mice brains—however, to increase the effectiveness of NP uptake, microbubble-assisted ultrasound-guided BBB disruption was utilised. They noted the peak inhibition of the tumour occurred around day 53 post-treatment commencement and had a median survival time of 80 days compared to 54 days, when the NP was delivered without focused ultrasound.

**Figure 5:**
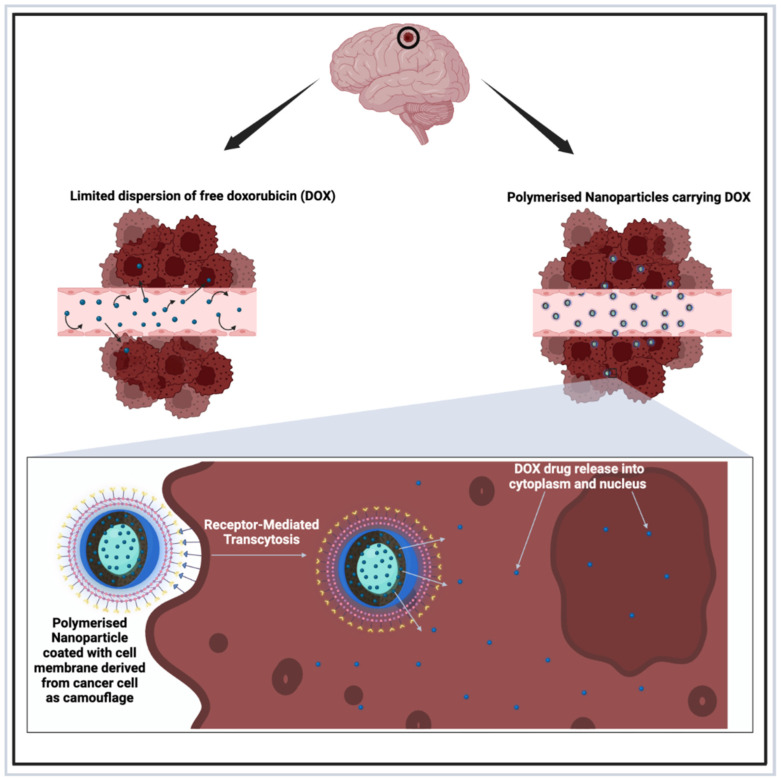
Receptor-mediated trancytosis can also be used to increase cancer cells’ toxicity. Such a Trojan-horse strategy can be used to facilitate the release of Doxorubicin (DOX) into the cytoplasm and nucleus by nanoparticles exploiting a mimicry coating based on cancer-cell-derived membranes. This strategy has been nicely described in breast-induced brain metastases by Liu et al. [111]. Created in BioRender. Khilar, S., 2025 (https://BioRender.com/m06x929/, last modified on 21 January 2025).

In neuro-oncology, the term “Trojan Horse strategy” is commonly used to describe the use of receptor-mediated transcytosis (see Figure 5); this approach can be mediated by transferrin (see above in Section 5.1.3 and Section 5.2.3) but also apolipoprotein [137] and photoactivated therapy candidates, such as cyclic ruthenium–peptide conjugates [138]. Liu et al. [111] referred to this masking strategy when they proposed the preparation of DOX-loaded polymeric NPs, which had a coating derived from the MDA-MB-231/Br cell membrane, “a brain homing MDA-MB-231 breast cancer cell”; collectively, their NPs were called DOX-PLGA@CM. Kaplan–Meier survival analyses showed that the systemic administration of these NPs significantly increased the survival of mice to 59 days compared to free DOX (48 days).

Finally, the development of mRNA vaccines is giving a new boost to the hopes of finding a long-lasting treatment for various cancers. These vaccines work by encoding tumour-specific antigens and immune-stimulating molecules, effectively activating the immune system to target and eliminate cancer cells [139].

With more than 120 clinical trials to date demonstrating their potential across various malignancies, including brain tumours, nanotechnology could play a pivotal role in improving them through the mechanisms described above, hence allowing to achieve more efficient delivery and precise regulation of the immune response.

### 5.3. Radio-Immunotherapy

Any type of radiation therapy in principle modulates the local tumour microenvironment (TME) of irradiated lesions, a property that can be exploited with the use of immunomodulators to enhance the therapeutic value of RT [141]. In this subsection, we will explore how various nanostrategies have been used to achieve enhanced efficacy in the management of primary and secondary brain tumours; specifically, we will cover the use of stem cells, vesicles, and nanostars, and we will describe how nanomedicine helps in harnessing them against cancer cells.

In recent years, we have witnessed an increasing interest in the utilisation of immunotherapy alongside RT and chemotherapy, particularly for the treatment of HGG. Growing tumour cells/masses can prevent the body’s immune cells from recognising or killing tumour cells by dysregulating signalling pathways and immunosuppressive cells or cytokines [142,143]; however, the recognition that the tumour microenvironment possesses immune privilege [144,145] led to proposing high-dose hypofractionated RT as a vital adjuvant to immunomodulatory therapy, particularly in occult metastases [141,146,147].

Going beyond primary brain tumours, Kiess et al. [148] combined Ipilimumab (Ipi) with SRS for the treatment of melanoma brain metastases and showed that the association between timing of SRS/Ipi and OS was statistically significant, and when patients received SRS during or before immunotherapy, OS was better and showed lesser recurrence of the tumour regionally. Hence, the combination of RT with immune checkpoint blockade immunotherapy has the potential to mount a robust immune response against tumour cells and potentially evade the issue of immune privilege in tumour tissue. Nonetheless, challenges still exist regarding the accurate targeting of immunomodulators to the tumour microenvironment despite synergising RT with immune checkpoint blockade therapy.

Wang et al. [149] attempted to solve this issue by encapsulating aPD-L1 and diselenide-bridged MSNs within a mesenchymal stem cell (MSC) membrane. CC chemokine receptor 2 (CCR2) was overexpressed on the MSC membrane. After irradiating the glioma tumour cells, the NPs could be directed toward the chemokine (CC motif) ligand 2 (CCL2), which is greatly expressed through radiation-induced tropism. In fact, the migration of the biomimetic nanoplatform designed in this study (which had greatly overexpressed CCR2 containing MSC), termed CCR2-SCM@MSN, was greatly improved towards the mouse glioma cell line GL261 that received X-ray irradiation pre-treatment (especially in comparison to cells that had not received X-ray irradiation).

When it came to the delivery of aPD-L1, CCR2-SCM@MSN nanoplatforms showed greater release of these antibodies and greater binding affinity when exposed to X-ray irradiation. Moreover, PD-L1 signals were substantially lost in X-ray-irradiated GL261 cells secondary to CCR2-SCM@MSN aPD-L1 exposure. Lastly, these biomimetic nanoplatforms also exhibited a reduction in immunotherapy-related adverse events by showing less colonisation with secondary antibodies in organs like spleen, kidneys, liver, lungs and heart.

The combination of immunotherapy with RT was also tested by Chen et al. [150] when they combined gold NPs with *E. coli*-derived outer membrane vesicles (OMVs), creating the complex Au-OMV. Gold NPs have numerous advantageous properties such as the ability to have numerous molecular surface coatings, biocompatibility, and ability to be synthesised into different sizes. Within the study by Chen et al., those NPs needed to have a concentration of 200 μg mL^−1^ and exposure to RT to exert cytotoxic effects.

This combination also reduced the survival rate of GL261 mouse glioma cell lines (alongside B.end3 mouse brain endothelial cells and C8D1A mouse astrocytes) from 80% to 30%. Cancer cell death with the generation of reactive oxygen species with the use of metal-based NPs such as gold was already known [151]; however, this study showed that with the creation of the Au-OMV complex, ROS generation in GL261 glioma cells increased by five-fold when RT was co-applied, compared to the control group, and approximately 2.5 times the amount when Au-OMV was utilised alone.

This shows that Au-OMV complexes can serve as important radiosensitisers, similarly to the plasmonic gold nanostars [50] mentioned earlier with regard to their use as optical imaging contrast, photoactivated transducer, and therapeutic agents.

### 5.4. Anti-Angiogenic Therapy

The high vascularisation of brain tumours in general and gliomas in particular is the fundamental reason why anti-angiogenic therapeutics have been extremely successful as adjuvant treatments. In this subsection, we will cover how nanotechnologies have been adopted to further increase the efficacy of anti-angiogenic treatments.

Two overexpressed receptors found on the surface of new blood vessels in gliomas are vascular endothelial growth factor 2 (VEGFR-2) and Neurolipin-1 (NRP-1). In 1977, the potent antiangiogenic protein Endostatin was identified and was shown to be able to systemically inhibit tumour growth and metastasis [152]. In 2020, Lu et al. [153] designed a modified version of the penetrating peptide-modified polyethyleneimine (PEI) nanocomplex to provide a potent and safe medium of gene delivery. This was carried out by combining PEI with a dual BBB-penetrating peptide TAT-AT7 (which had been created by attaching the cell-penetrating peptide TAT to a vascular-targeting peptide AT7, to target binding to the VEGFR-2 and NRP-1) with the goal of improving the binding affinity and BBB/BTTB crossing capacity of the entire nano-complex. The combination of PEI and TAT-AT7 was termed PPTA by the study’s authors, and the nanocomplex was then loaded with pVAXI-En plasmid to create PPTA/pVAXI-En. The pVAXI-EN was the secretory endostatin gene, which inhibits angiogenesis. In comparison to AT7 and TAT alone, the combination achieved a 3–10-fold greater binding affinity to VEGFR-2 and NRP-1 and exhibited a 119-fold greater endothelial cell uptake compared to AT7 alone.

## 6. Discussion

This scoping review allowed us to cover the vast literature on nanodrugs with the current or forecasted scope in the management of primary and secondary brain tumours. With the rapid development of delivery systems at the nano scale, consisting of either organic or inorganic nanocarriers, such as nanoshells, micelles, liposomes, and nanoparticles, it has been possible to tackle selective cancer targets relevant to neuro-oncologists. Various types of mechanisms, ranging from those to enhance BBB permeability to applications in thermotherapy, immunotherapy, and radio-immunotherapy against cancer cells, have been presented, along with the rationale for their testing in vivo and in vitro. The summary of the evidence collected through our review of the literature has then been structured in Section 5.1, Section 5.2, Section 5.3 and Section 5.4 with the aim of providing guidance through this complex area of nanomedicine.

A few take-home messages should be listed.

(a) The scenarios presented illustrate the different stages of readiness, with some solutions that are already being tested in patients and others that are far too premature despite promising laboratory results.
(b) This scoping review outlines some commonalities between primary and secondary brain tumours, commonalities which can be exploited by scientists to identify innovative solutions and change the way we diagnose and treat patients with brain tumours. Furthermore, it highlights the bottlenecks of current management, from barriers to vehiculate contrast agents and drugs across the BBB and BTB to the issue of the tumour microenvironment’s immune privilege [92,144,145], from metabolic plasticity for brain metastases [70,154] to the issue of nanotoxicity.
(c) We found a rising interest regarding the link between different types of primary tumours and ways to target common aspects of their biology. For instance, regarding the association between malignant melanoma (MM) and GBM, we counted fifteen studies with a total of 220 patients who all showed an association between these two tumour types [121]. Analysing those studies in detail, several mechanisms to support this linkage and possible targets for therapeutic solutions were noted, such as telomerase reverse-transcriptase promoter mutations [122,123,124,125,126,127], protein tyrosine phosphate receptor type D gene mutations occurring at high rates [128], and BRAF mutations [129,130,131,132]. Interestingly, all of them have been tested using various immunotherapy strategies [134,135,136], indicating that this area requires closer inspection and research, especially due to the aggressive nature of brain tumours.

It is therefore clear that various types of nanoconstructs possess specific advantages that make each of them potential valuable additions to our therapeutic armamentarium. For instance, we highlighted the ability of polymeric NPs to target brain metastases and their primary tumour, a strategic advantage that could possibly open the doors to the management not only of metastatic patients but also of those with more than one primary cancer, show are unfortunately on the rise in many worldwide statistics. We outlined how various NPs have multifold actions, from HA’s ability to encapsulate contrast media and shield chemotherapeutic drugs to the specific use of magnetic NPs in adjuvant treatment thanks to their ability to be controlled remotely after administration. Furthermore, we pointed out how nanomedicine allowed diagnostic applications to be converted for theranostic purposes, moving from the intratumoural uptake of iron oxide (magnetite) NPs to their use in thermotherapy protocols (by alternating magnetic fields to provide particle heating) and fractionated SRS. These examples demonstrate not only the potential for enhancing the medical but also the surgical management of neuro-oncology patients described at the beginning of this article. All these aspects indicate a golden trend in nanomedicine, which is the tendency to identify new solutions to older problems and adopt them to exponentially increase the therapeutic options in a range of clinical scenarios.

As much as NPs often exploit their potential bioavailability and biomimicry, overall nanotoxicity remains, at present, the biggest limiting factor for the development and further application of innovative nanosolutions. Of note, the effective drug delivery provided by coating of NPs in PEG solutions (as presented in Section 5.1.2 and Section 5.1.5) does not come without downsides: on one hand, this solution lacks long-term colloidal stability; on the other hand, it also exhibits high nonspecific toxicity to BBB endothelial cells. Hence, despite the advantage of providing an accelerated intratumoural chemotherapy accumulation, PEGylation strategies still suffer from a potentially detrimental iatrogenic risk. Similar risks of nanotoxicity are particularly noticeable when reviewing various NPs used for immunotherapy or as radiosensitisers. Some of these mechanisms of nanotoxicity can be unforeseeable; others can only be prevented by fine-tuning their administration. From a biological perspective, nanotoxicity can occur at the genomic (damage to the DNA per se) and/or epigenomic (alteration of the chemical and enzyme mediated processes that up- or down-regulate gene expression) level(s) and involves various direct and indirect mechanisms such as oxidative stress, inflammatory changes, the alteration of DNA replication, transcription and repair, hypoxia, the impairment of DNA methylation, histone modification, and damage to noncoding RNAs [155]. For instance, with regards to the immunotherapy strategies presented above for immune checkpoint receptors, their inherent risk consists in exacerbating immune-related adverse events due to their non-specific and systemic in vivo distribution. On the contrary, with MNPs, a precise oversight on the setting of the magnetic field (the stronger it becomes, the greater the chances of these magnetic NPs attracting one another, aggregating and causing emboli) will suffice to avoid complications.

As such, a caveat common to all the nanostrategies described above consists of the attention that should be paid by the neuro-oncological community to understand which mechanisms of actions are at play and to what extent they can generate unintended iatrogenic nanotoxicity before translating their use from laboratory settings to widespread adoption in day-to-day clinical care.

### Limitations of the Study

This study has several limitations, partly due to the nature of a scoping review (which is to monitor the body of evidence on a large topic) but also related to our decision to not limit our search study to research conducted on humans. While this allowed us to describe the impact of nanosolutions on various types of tumour models, which was in keeping with our aim to review the literature on primary and secondary brain tumours, it created some heterogeneity, which we were only able to partially address when we summarised the results of our search. In practice, while the first aim of our scoping review has been achieved (to identify hot topics and emerging trends in the utilisation of nanomedicine in drug delivery for primary and secondary brain tumours), the information available to achieve the second aim (to demonstrate whether and how nanomedicine is extending survival and quality of life in patients diagnosed with primary or secondary brain tumours) was too patchy for us to achieve a conclusion. This knowledge gap justifies why we managed to explore the efficacy (explaining how and why a treatment strategy works in an experimental setting), but we failed to provide additional details regarding the effectiveness (how well a treatment strategy improves outcomes in real-life scenarios) of those nanostrategies. The ongoing development of nanomedicine discouraged us from defining strict timeframes for our search, and this increased even further the volume of articles triaged for inclusion in this scoping review. Hopefully, in our future studies, we will be able to proceed with more specific systematic review questions such as the impact of NPs on selected aspects of neurosurgical practice (e.g., intraoperative imaging, adjuvant treatment and prognostication, etc.).

## 7. Conclusions

Overall, this scoping review focused on identifying studies that tested and recognised the ability of NPs as potent delivery systems for antineoplastic agents that can overcome the notorious hurdle of the BBB and potentially provide a means to prolong survival in patients with CNS tumours. The results discussed indicate that the study selected for analysis focused on a variety of nanometric products aimed at use in chemotherapy, immunotherapy, anti-angiogenic therapies, and RT. These studies allowed us to evaluate, understand, and reflect on the similarities many of these strategies have, including the receptors that various NPs target, the antineoplastic agents these NPs tend to be loaded with, and the cautious strategies used to combine them all (such as for radio-immunotherapy, where a synergistic effect at delivering treatment across the BBB results in prolonging patients’ survival). What we noted, however, was that several studies identified in this review worked on cell lines rather than actual human trials. This is not unexpected, considering that we did not restrict our search to translational studies but included all those with potential for future applications in human trials.

We, as authors, also acknowledge a key gap in the field—the difficulty in achieving a synergistic utilisation of several modalities into a single NP—because the tumour microenvironment possesses its immune privilege. Perhaps in the future, more studies will evaluate the plausibility of encapsulating chemotherapy agents with other immunotherapy and anti-angiogenic drugs within a single NP. Such an ideal NP could also have several specific antibodies on its outer layer to specifically target a large number of tumoural receptors.

## Figures and Tables

**Table 1:** Summary of the studies identified which used NPs as treatment modalities for primary brain tumours. They have been divided according to the NPs serving as a delivery system for chemotherapy, immunotherapy, radio-immunotherapy, and anti-angiogenic therapy. Their administration models are also listed according to whether they were HT—a human trial; EM—an experimental model; in vivo; in vitro.

Treatment Modality Using NPs: Primary Brain Tumours	Reference	Administration Model	Strategy Described in the Study
Chemotherapy	Maier-Hauff et al. [60]	HT	Applied Intratumoural Thermotherapy using iron oxide (magnetite) NPs and alternating magnetic field (AMF).
	Liu et al. [63]	EM—in vivo (cultured C6 tumour cells) and in vitro	Combined FUS and MNPs (encapsulated iron oxide (Fe_3_O_4_) within poly [aniline-co-N-(1-one-butyric acid)] aniline (SPAnH) as a surface layer).
	Janjua et al. [66]	EM—in vivo (U87 and GL261 glioblastoma cell lines) and in vitro	Developed novel ultra-small (30 nm) Silica Nanoparticles for the delivery of TMZ across the BBB.
	Wan et al. [69]	EM—in vivo (Glioma cells of U251, BMSCs, HUVECs, SHG44 and U87 lines) and in vitro	Used NPs within a Zirconium-based framework to deliver TMZ with the concurrent use of ultrasound,
	Chang et al. [54]	EM—in vivo (GL261 glioma cells) and in vitro	Conjugated cisplatin with Pluronic F127-complexed PEGylated poly(glutamic acid) to produce an NP called PLG-PEG/PF127-CDDP.
	Ao et al. [57]	EM—in vivo (U87 MG cell line)	ACG-loaded nanomicelles in three different feeding ratios, ACGs/EB5-NCs, ACGs/EB10-NCs, and ACGs/EB20-NCs, were delivered using Poly(ethylene oxide)-b-poly(butylene oxide) (PEO-PBO), as an amphiphilic polymeric carrier toward U87 MG tumour-bearing mice. The NPs had the following sizes: 148.8 ± 0.5 nm, 32.7 ± 4.1 nm, and 27.1 ± 0.3 nm, corresponding to ACGs/EB5-NCs, ACGs/EB10-NCs and ACGs/EB20-NCs, respectively.
Immunotherapy	Galstyan et al. [80]	EM—in vivo (Mouse glioblastoma cell line GL261 implanted intracranially in 8 weeks old female C57BL/6J mice)	Abx against CTLA-4 and PD-1 was covalently bonded to a drug carrier called the poly (Beta-L-malic acid) PMLA backbone.
	Zhang et al. [81]	EM—in vivo (orthotopic GBM-bearing mice)	Loaded antibodies against PD-1 (as termed by the study aPD-L1) into redox-responsive micelles and combined it with Paclitaxel (PTX).
Radio-immunotherapy	Wang et al. [149]	EM—2 murine models with orthotopic GBM tumours used	Encapsulated PD-L1 antibodies (alphaPD-L1) and diselenide-bridged mesoporous silica nanoparticles (MSNs) within a mesenchymal stem cell (MSC) membrane. CC chemokine receptor 2 (CCR2) was also overexpressed on the MSC membrane. Glioma tumour cells were concurrently irradiated, which allowed radiation-induced tropism of NPs towards chemokine (CC motif) ligand 2 (CCL2).
	Chen et al. [150]	EM—*E. coli* cells and GL261 mouse glioma cells, C8D1A mouse astrocytes, B.end3 mouse endothelial cell lines and RAW264.7 mouse macrophages	Combined gold NPs (AuNP) with an outer membrane vesicle (OMV) derived from E.Coli to create the Au-OMV complex. The complex increased ROS generation in GL261 glioma cells by 2.5-fold when they were treated with RT compared to just the Au-OMV complex alone.
Anti-angiogenic therapy	Lu et al. [153]	EM—in vivo (Orthotopic U87-mCherry-luc glioma-bearing nude mice) and in vitro	Penetrated peptide-modified polyethyleneimine (PEI) nanocomplex with TAT-AT7 on the surface to improve binding and crossing BBB. The nanocomplex was loaded with the pVAXI-EN plasmid (secretory endostatin gene)—the total complex was termed PPTA/pVAXI-En.

**Table 2:** Summary of the studies identified which used NPs as treatment modalities for secondary brain tumours. They have been divided according to the tumour’s primary site and NPs serving as a delivery system for chemotherapy, immunotherapy, immunotherapy + SiRNA, EGFR-tyrosine inhibitors, and radio-immunotherapy. Their administration models are also listed according to whether they were HT—a human trial; EM—an experimental model; in vivo; in vitro.

Treatment Modality Using NPs: Secondary Brain Tumours		Reference	Administration Model	Strategy Described in the Study
Chemotherapy	Breast	Lim et al. [100]	EM—n vivo (brain metastases bearing mouse model) and in vitro (BT474 cells breast cancer cell lines)	Loaded hyperbranched polymers (HBPs) with Doxorubicin (DOX) and labelled the NP with anti-HER3/anti-PEG bispecific-antibody fragments (HER3-HBP-DOX) group.
	Breast	Ashokan et al. [70]	EM—MDA-MB-231 breast cancer cell line, MDA-MB-231-BR and Breast cancer cell line HCC1806 used.	Loaded NP with a combination of Platin-M (cisplatin prodrug) and a glycolysis inhibitor to simultaneously target the primary tumour site and tumour cells that had metastasised to the brain (the potential advantages of using glycolysis inhibitors were highlighted by [112,113]).
	Breast	Liu et al. [111]	EM—in vivo (brain metastases breast cancer model)	“Trojan Horse strategy,”—a polymeric NP had a coating derived from the MDA-MB-231/Br cell membrane and was loaded with Doxorubicin. Collectively called DOX-PLGA@CM.
Immunotherapy	Breast	Sevieri et al. [107]	EM—in vitro (using D2F2/E2-Luc cells) and in vivo (murine breast tumour cell line D2F2/E2, that expressed human HER2 receptor)	Combined Transtazumab with Ferritin NPs and Docetaxel (H-TZ + Dtx) for targeted drug delivery within the tumour microenvironment and for aiding the composition of a protective microenvironment against tumour cells.
Immunotherapy + siRNA	Breast	Ngamcherdtrakul et al. [110]	EM—in vivo (drug-resistant orthotopic HER2+ HCC1954 tumour mouse model and HER2+ BT474 tumours within mice brains)	Co-delivery of Docetaxel and HER2 targeting siRNA via a trastuzumab-conjugated NP towards the HER2 + HCC1954 drug-resistant tumour mouse cell line.
Chemotherapy	Lung	Sambade et al. [132]	EM—in vivo (intracranial A549 tumours in nude mice)	Docetaxel and acid-labile C2-dimethyl-Si-Docetaxel (C2-Docetaxel) were carried in “Particle Replication in Nonwetting Templates (PRINT(^®^)) PLGA” NPs. Within A549 tumours in nude mice, median survival was seen to have increased by 35% when PRINT-C2-Docetaxel was used.
siRNA delivery	Lung	Zhang et al. [115]	EM—in vivo (mice bearing SCLC tumour metastasis model) and in vitro studies	Designed an NP capable of targeting tumour cells which had metastasised to the brain from small cell lung cancer (SCLC)—the incidence of brain metastases from SCLC is 40–50% in advanced stages of SCLC and 10% in early stages [114]. Called TP-M-Cu-MOF/siATP7a, the NP was loaded with siRNA targeting the ATP7a gene, which is important in modulating the efflux of copper intracellularly. The NP had a coating made of the TP0751-peptide-decorated stem cell membrane, which was syphilis-derived as Pallidum can traverse the BBB [120], and had a copper-based framework. Overall, the NP took advantage of cupropoptosis to inhibit tumour cell growth [116,117,118,119].
EGFR-tyrosine kinase inhibitors	Lung	Kim et al. [133]	EM—in vivo (Human NSCLC cell lines (HCC827 and H1975) and HCC827-luc cells implanted into xenograft mouse models	NUFS-sErt—a water-soluble NP designed using fat and supercritical fluid which delivered Osimertinib (a third-generation EGFR–tyrosine kinase inhibitor) for the treatment of EGFR-mutant lung cancer. This was carried out to counteract the problem of poor solubility of Osimertinib, which has been shown to have significantly higher brain penetration [138]. Significant tumour growth inhibition was seen when NUFS-sErt was inserted into the brain ventricle in intracranial xenograft model.
Radio-immunotherapy	Lung, Breast, Melanoma and Colon	Verry et al. [71]	HT—Phase I	Phase I NANO-RAD trial showing the use of a gadolinium-based NP in combination with radiotherapy for the treatment of brain metastases from breast, lung, melanoma and colon cancer.

## Data Availability

No new data were created by the authors of this scoping review, which has solely summarised the existing evidence from the scientific literature on nanomedicine.

## Abbreviations

ACGs	Annonaceous acetogenins
AGuIX	Activation by the guidance of irradiation by X-ray
AMF	Alternating magnetic field
BBB	Blood–brain barrier
BTB	Blood–tumour barrier
CCL2	Chemokine (CC motif) ligand 2
CCR2	CC chemokine receptor 2
CNS	Central nervous system
CPPs	Cell-penetrating peptides
CTLs	Cytotoxic T-lymphocytes
CTLA-4	Cytotoxic T-lymphocyte-associated antigen 4
DAMPs	Damage-associated molecular patterns
DCA	Dichloroacetate
DOX	Doxorubicin
ECM	Extracellular matrix
EMF	External magnetic field
EOR	Extent of resection
EPR	Enhanced permeability and retention
fMRI	Functional magnetic resonance imaging
FUS	Focused ultrasound
GBM	Glioblastoma
HA	Hyaluronic acid
HACE	Hyaluronic acid–ceramide
HA-NPs	Hyaluronic acid nanoparticles
HBP	Hyperbranched polymers
HER	Human epidermal growth factor receptor
HFn	Ferritin nanoparticles
HGGs	High-grade gliomas
iCT	Intraoperative computed tomography
IN	Intraoperative neurophysiology
IoUS	Intraoperative ultrasound
Ipi	Ipilimumab
LGG	Low-grade gliomas
mAbs	Humanised monoclonal antibodies
MGMT	O-methylguanine-DNA methyltransferase
MM	Malignant melanoma
MNPs	Magnetic nanoparticles
MOFs	Metal–organic frameworks
MRI	Magnetic resonance imaging
MRP1	Multi-drug resistant protein 1
MSC	Mesenchymal stem cell
MSNs	Mesoporous silica nanoparticles
NICs	Nanoscale immunoconjugates
NMRSA	Proton magnetic resonance spectroscopic imaging
NPs	Nanoparticles
NRP-1	Neurolipin-1
o-HA	Hyaluronic acid oligomers
OMV	Outer membrane vesicle
OS	Overall survival
PD-1	Programmed cell death-1
PEI	Polyethyleneimine
PEO-PBO	Poly(ethylene oxide)-b-poly(butylene oxide)
PFS	Progression-free survival
P-gp	P-glycoprotein
PTX	Paclitaxel
RT	Radiotherapy
SCLC	Small-cell lung cancer
SLNs	Solid lipid nanoparticles
SRS	Stereotactic radiosurgery
TMZ	Temozolomide
TfR1	Transferrin receptor 1
Tregs	Regulatory T-cells
TME	Tumour microenvironment
TPP	Triphenylphosphonium
TTPs	Tumour-targeting peptides
TZ	Trastuzumab
USLPs	Ultra-small Silica NPs with large pores
usNLCs	Ultra-small nanostructure lipid carriers
VEGFR-2	Vascular endothelial growth factor 2
WHO	World Health Organisation
